# Functional polarization of human hepatoma HepaRG cells in response to forskolin

**DOI:** 10.1038/s41598-018-34421-8

**Published:** 2018-10-31

**Authors:** Abdullah Mayati, Amélie Moreau, Marc Le Vée, Arnaud Bruyère, Elodie Jouan, Claire Denizot, Yannick Parmentier, Olivier Fardel

**Affiliations:** 1 Univ Rennes, Inserm, EHESP, Irset (Institut de recherche en santé, environnement et travail) - UMR_S 1085, F-35000 Rennes, France; 2 Centre de Recherche en Pharmacocinétique, Technologie Servier, F-45000 Orléans, France; 3 Pôle Biologie, Centre Hospitalier Universitaire, F-35033 Rennes, France

## Abstract

HepaRG is an original human hepatoma cell line, acquiring highly differentiated hepatic features when exposed to dimethylsulfoxide (DMSO). To search alternatives to DMSO, which may exert some toxicity, we have analyzed the effects of forskolin (FSK), a cAMP-generating agent known to favor differentiation of various cell types. FSK used at 50 µM for 3 days was found to promote polarization of high density-plated HepaRG cells, *i.e*., it markedly enhanced the formation of functional biliary canaliculi structures. It also increased expressions of various hepatic markers, including those of cytochrome P-450 (CYP) 3A4, of drug transporters like NTCP, OATP2B1 and BSEP, and of metabolism enzymes like glucose 6-phosphatase. In addition, FSK-treated HepaRG cells displayed enhanced activities of CYP3A4, NTCP and OATPs when compared to untreated cells. These polarizing/differentiating effects of FSK were next shown to reflect not only the generation of cAMP, but also the activation of the xenobiotic sensing receptors PXR and FXR by FSK. Co-treatment of HepaRG cells by the cAMP analog Sp-5,6-DCl-cBIMPS and the reference PXR agonist rifampicin reproduced the polarizing effects of FSK. Therefore, FSK may be considered as a relevant alternative to DMSO for getting polarized and differentiated HepaRG cells, notably for pharmacological and toxicological studies.

## Introduction

The hepatic bi-progenitor cell line HepaRG is an original human hepatoma cell line, that presents itself as a mixture of hepatocyte islands and cholangiocyte-like cells when cultured in appropriate conditions, *i.e*., in the presence of the differentiating agent dimethyl sulfoxide (DMSO)^1^. Cultures of confluent HepaRG cells exposed to 2% (vol/vol) DMSO for 15 days exhibit key hepatic hallmarks, including high functional expression of drug detoxifying enzymes and drug-sensing nuclear receptors, like pregnane X receptor (PXR) and constitutive androstane receptor (CAR)^2^. DMSO concomitantly promotes the polarization of HepaRG cells forming hepatocyte islands, with the appearance of functional bile canaliculi (BC) and expression of main drug transporters at sinusoidal and apical poles of cells^3^. Such data thereby support the increasing relevant use of DMSO-treated HepaRG cells for drug detoxification and/or toxicity studies^4–6^.

The mechanisms by which the non-physiological agent DMSO maintains *in vitro* liver-specific differentiated functions are presently largely unknown^7^. Moreover, DMSO exerts some toxicity towards HepaRG cell cultures^8^ and can affect or interfere with hepatic functions, including some unrelated to drug metabolism^9^. Alternatives to the use of DMSO for obtaining polarized differentiated HepaRG cells are therefore interesting to identify and characterize. In this context, culturing HepaRG cells in a three-dimensional environment or under hyperoxic conditions may help to generate polarized HepaRG cells displaying substantial hepatic functions without, or with reduced concentrations, of DMSO^10,11^. Similarly, overexpression of CAR, a nuclear receptor controlling various drug metabolism genes and acting as a key regulator for the hepatic differentiation and maturation of human embryonic stem cells (hESCs)^12^, has been recently shown to enhance the differentiation of HepaRG cells, in the absence of DMSO, creating thus a physiologically relevant environment for studies on hepatic drug metabolism^13^. Among potential additional alternatives to DMSO for getting differentiated/polarized HepaRG cells, the natural cAMP elevating compound forskolin (FSK) has likely to be considered. Indeed, this diterpene, which directly activates the adenylate cyclase enzyme to generate cAMP from ATP^14,15^, is known to induce differentiation in various cell types^16,17^ and to trigger and/or enhance polarization of rodent hepatocytes and human hepatoma HepG2 cells^18,19^. Moreover, cAMP has been recently demonstrated to promote the maturation of human pluripotent stem cell-derived hepatocytes^20^. The present study was therefore designed to analyze the effects of FSK on polarization and differentiation of HepaRG cells. Our data demonstrate that the natural diterpene stimulates the formation of functional BC in HepaRG cell culture, likely in a cAMP/PXR-dependent manner.

## Materials and Methods

### Chemicals and reagents

FSK, 1,9-dideoxyforskolin (DDF) and GW4064 were from Santa Cruz Biotechnology (Heidelberg, Germany). N^6^-Benzoyladenosine-3′,5′-cyclic monophosphate (6-Bnz-cAMP) and acetoxymethyl ester form of 8-(4-chlorophenylthio)-2′-*O*-methyl-cAMP (8-pCPT-cAMP) were from Tocris (Bristol, United Kingdom). 5, 6- Dichlorobenzimidazole riboside- 3′, 5′- cyclic monophosphorothioate, Sp- isomer (Sp-5,6-DCl-cBIMPS) was supplied by BIOLOG Life Science Institut (Bremen, Germany). Rifampicin, nifedipine, probenecid, diclofenac, 8-bromoadenosine cAMP (8-Br-cAMP), 5(6)-carboxy-2, 7-dichlorofluorescein (CF) diacetate and 3-isobutyl-1-methylxanthine (IBMX) were provided by Sigma-Aldrich (Saint-Quentin Fallavier, France). Hydrocortisone hemisuccinate was supplied by SERB laboratories (Paris, France). [^3^H(G)] taurocholic acid (specific activity = 1.19 Ci/mmol), and [6,7-^3^H(N)] estrone-3-sulfate (E3S) (specific activity = 57.3 Ci/mmol) were purchased from Perkin-Elmer (Courtaboeuf, France). All other chemicals and reagents were from the highest available grade. Chemicals were commonly used as stock solutions in DMSO. Control cultures received the same dose of solvent, not exceeding 0.2% (vol/vol), than treated counterparts.

### Cell culture

Wild-type HepaRG cells were cultured in Williams’ E medium supplemented with 10% (vol/vol) fetal calf serum, 20 IU/mL penicillin, 20 µg/mL streptomycin, 5 µg/mL bovine insulin, 2 mM glutamine, and 50 µM hydrocortisone hemisuccinate. Cells were routinely passaged through plating them at low density (27 000 cells/cm^2^), allowing to reach confluency after 3–5 days of culture, and culturing them for two weeks before trypsin treatment and re-seeding. Conventional DMSO-mediated differentiation of HepaRG cells was performed through culturing cells for two weeks in the absence of DMSO, following by two additional weeks in the presence of 2% (vol/vol) DMSO, in order to promote expression of differentiated hepatic markers and functions, as previously described^1^. Experiments with FSK and chemicals were mainly done with HepaRG cells from passages 13–17, plated at high density (200 000 cells/cm^2^), to obtain near confluency after an initial 4–6 h period of cell plating, from which FSK/chemical treatments were usually initiated. PXR-knockout (KO) HepaRG cells and parental control 5 F HepaRG cells, supplied by Sigma-Aldrich, were cultured as reported above for wild-type counterparts. Freshly isolated human hepatocytes were obtained by enzymatic dissociation of histologically-normal liver fragments from four donors undergoing hepatic resection for tumors, via the Centre de Ressources Biologiques Santé of Rennes BB-0033-00056, which has obtained the authorization N°DC-2008-630 from the French Ministry of Health to collect hepatic resections from the digestive surgery department and then to isolate and deliver the hepatocytes used in this study. All of liver fragment donors were adult and provided a written informed consent to participate in the study. All experimental procedures complied with French laws and regulations; they were approved by the local institutional ethics committee (Pontchaillou Hospital, Centre Hospitalier Universitaire, Rennes, France).

### Quantification of BC-like structures

BC formed in HepaRG cell cultures appear as characteristic refractive structures, corresponding to bright/white objects on phase-contrast photographs, as previously reported^21^, whereas cells appear as dense/black elements, containing nuclei recognizable by their round/delimited shape and differential density. After capturing images using an Axiovert 40 C phase contrast microscope (Carl Zeiss, Le Pecq, France) and adjusting brightness parameters to optimally distinguish between white and black densities, bright objects reflecting BC as well as round/delimited nuclei-related objects were segmented by adjusting the shape and area parameters to exclude non-corresponding objects, using a dedicated in-house macro-program based on ImageJ 1.48 software^22^. BC numbers and areas as well as nuclei numbers were next determined from 3 zones per condition counting from at least 3 independent experiments. In some experiments, BC were additionally visualized through immuno-labelling of BC with anti-P-glycoprotein (P-gp) or anti-multidrug resistance-associated protein (MRP) 2 antibodies, as reported below, and their number was quantified using ImageJ and relatively to 4′,6-diamidino-2-phenylindole (DAPI)-stained nuclei.

### RNA isolation and analysis

Extraction of total mRNA from cells was performed using the TRIzol reagent (Invitrogen, Cergy-Pontoise, France). RNA (10 ng) was then subjected to reverse transcription-quantitative polymerase chain reaction (RT-qPCR), using the fluorescent dye SYBR Green methodology and a CFX384 detector (Bio-Rad, Marnes-la-Coquette, France)^23^. Primers were specific ready-to-use KiCqStart® gene primers (from Sigma Aldrich) or in-house custom primers, whose sequences are indicated in Supplementary Table S1. Amplification curves of the PCR products were analyzed with the CFX Manager software (Bio-Rad), using the comparative cycle threshold method. Relative quantification of the steady-state target mRNA levels was calculated after normalization to the 18S rRNA level, used here as an internal control. Data were expressed as % of expression levels found in isolated human hepatocytes, arbitrarily set at 100%, or as fold changes relative to mRNA levels in control cells.

### RNA interference experiments

HepaRG cells were plated at high density (200 000 cells/cm^2^), in the presence of 100 nM siRNAs against PXR (SiPXR) (Sigma Aldrich) or of non-targeting siRNAs (SiNT) (Sigma Aldrich), used as a control. Transfections were performed using the transfecting reagent Lipofectamine™ RNAiMAX, according to manufacturer instructions (Thermo Fisher Scientific, Villebon sur Yvette, France). After 24 h, transfection medium was discarded and cells were exposed to FSK for 72 h.

### Western-blot analysis

Cellular protein extracts, prepared from HepaRG cells as previously described^24^, were separated on polyacrylamide gels and electrophoretically transferred to nitrocellulose membranes. Gel loading and transfer were checked by staining membranes with the red dye Ponceau S. After blocking in Tris-buffered saline containing 4% bovine serum albumin, membranes were incubated overnight at 4 °C with mouse monoclonal primary antibody directed against PXR (clone H-11) (provided by Santa Cruz Biotechnology). Secondary peroxidase-conjugated monoclonal antibody was thereafter used to detect the primary antibody. After washing, immunolabeled proteins were finally visualized by chemiluminescence.

### Immunocytochemistry

Immunocytochemistry analyses were performed as previously described^11^. Briefly, wild-type, F5 or PXR-KO HepaRG cells were plated on 8-well chambered glass Lab-TEK™ systems (Thermo Fisher Scientific). After treatment, cells were rapidly washed by phosphate-buffered saline (PBS) and then fixed in ice-cold acetone for 10 min. Cells were then incubated for 1 h with 4% bovine serum albumin/PBS solution, before being incubated over-night at 4 °C with primary monoclonal antibodies raised against P-gp (clone C219), MRP2 (clone M2III-6), cytochrome P-450 (CYP) 3A4 (clone HL3) or albumin (clone F-10) (purchased from Alexis Biochemicals, Lausen, Switzerland, or from Santa Cruz Biotechnology) or with primary rabbit polyclonal antibody anti-zona occludens-1 (ZO-1) (provided by Abcam, Cambridge, United Kingdom). After washing in PBS, cells were subjected to 1 h-incubation with Alexa Fluor-coupled secondary antibody (Cell Signaling, Leiden, The Netherlands); nuclei were stained by incubation with DAPI for 5 min. Finally, a confocal fluorescence microscope LEICA DMI 6000 CS (Leica Microsystemes SAS, Nanterre, France) was used to acquire immunofluorescence images.

### Transport assays

Activities of the sinusoidal transporters sodium-taurocholate cotransporting polypeptide (NTCP) and organic anion transporting polypeptides (OATPs) were determined through measuring sodium-dependent-intracellular accumulation of the NTCP substrate taurocholate and probenecid-sensitive uptake of the OATP substrate E3S, as previously described^25^. In brief, HepaRG cells were incubated with radiolabeled substrates for 5 min at 37 °C, in a well-defined transport assay buffer consisting of 5.3 mM KCl, 1.1 mM KH_2_PO_4_, 0.8 mm MgSO_4_, 1.8 mM CaCl_2_, 11 mM D-glucose, 10 mM HEPES, and 136 mM N-methyl-D-glucamine (sodium-free buffer) or 136 mM NaCl (sodium-containing buffer) and adjusted to pH 7.4. After washing in PBS, cells were lysed, and accumulation of radiolabeled substrates was determined through scintillation counting and normalized to total protein content, determined by the Bradford method^26^. Taurocholate accumulation in the presence of sodium minus accumulation in the absence of sodium and E3S uptake in the absence of probenecid minus uptake in the presence of probenecid are thought to correspond to NTCP and OATP activities, respectively^25^.

MRP2 activity was studied through visualizing MRP2-mediated secretion of the fluorescent dye CF in BC lumen, as previously reported^3^. Briefly, HepaRG cells were incubated with 3 μM CF diacetate for 10 min at 37 °C; cells were then washed with ice-cold PBS and visualized using a Leica DM IRB microscope (Leica Microsystems) equipped with a black/white CoolSNAP ES camera (Roper Scientific, Planegg/Martinsried, Germany). Pictures were processed using the MetaMorph software (Molecular Devices, San Jose, CA, USA).

### Cytochrome P-450 activities

CYP3A4 and CYP2C9 activities were measured through analyzing oxidation of specific substrates, *i.e*., midazolam and diclofenac, respectively, using liquid chromatography coupled to tandem mass spectrometry (LC-MS/MS). Briefly, HepaRG cells were exposed to 100 µM midazolam or 100 µM diclofenac for 2 h in MEM medium, in the absence of fetal calf serum. Production of CYP activities-related metabolites, *i.e*., 1′-hydroxymidazolam and 4-hydroxydiclofenac, was then analysed in culture supernatants through LC-MS/MS-based analysis, using an high-performance liquid chromatography Aria system (Agilent, Les Ulis, France), equipped with a Kromasyl^®^ C18 (4.6 × 150 mm) column (Interchim, Montluçon, France) and coupled to a tandem mass spectrometry TSQ Quantum Ultra (Thermo Fisher Scientific) fitted with an electrospray ionization source (ESI+). Monitored ion transitions were at 342.0 > 168.0 m/z for 1′-hydroxymidazolam and 312.0 > 266.0 m/z for 4-hydroxydiclofenac. Amounts of produced metabolites were finally normalized to total protein cell content.

### Cellular cAMP quantification

Cellular cAMP levels were analyzed using the cAMP-Screen direct chemiluminescent competitive immunoassay system (Thermo Fisher Scientific), according to the instructions of the supplier.

### Statistical Analysis

Quantitative data were usually expressed as means ± S.E.M. Data were statistically analyzed using Student’s t test or analysis of variance (ANOVA) followed by the Dunnett’s or the Tukey’s post-hoc test. The criterion of significance was p < 0.05. Half maximal effective concentration (EC_50_) value for FSK-mediated stimulation of BC number was determined through nonlinear regression using Prism 5.0 software (GraphPad, San Diego, CA, USA).

## Results

### Stimulation of BC formation by FSK

**Figure 1:**
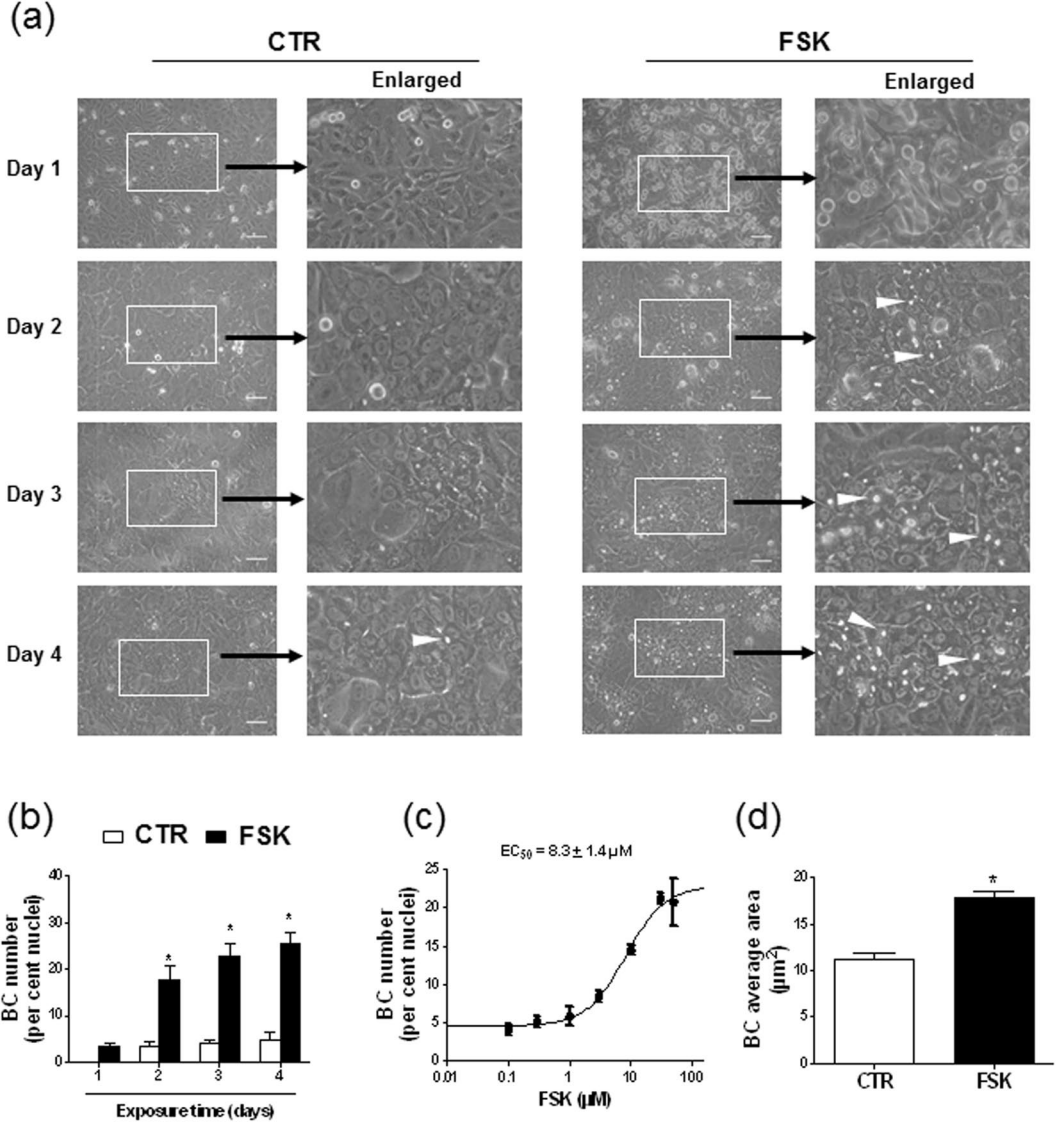
Formation of refractive BC-like structures in HepaRG cells exposed to FSK. HepaRG cells plated at high density were either exposed to 0.1% (vol/vol) DMSO (control/CTR) or treated for (**a**,**b**) 1, 2, 3 or 4 days or (**c**,**d**) 3 days by FSK, used at (**a**,**b**,**d**) 50 µM or (**c**) various concentrations (from 0.1 to 50 µM FSK). (**a**) Cells were then observed by phase contrast microscopy, allowing to distinguish refractive bright/white BC, indicated by white arrows on phase-contrast microscopic pictures; white bar = 50 µm. BC numbers (**b**,**c**) or area (**d**) were next determined by image analysis, as indicated in Methods. Data shown are representative of 4 independent experiments (**a**) or are the means ± SEM of at least 3 independent assays (**b**–**d**). (**b**,**d**) *p < 0.05 when compared to control cells. (**c**) FSK EC_50_ value towards BC formation is indicated at the top of the graph.

To investigate the potential effects of FSK on BC formation, HepaRG cells, seeded at high density, were exposed to 50 µM FSK and the morphological appearance of refractive BC was monitored by microscopic analysis over a 4-days period. The concentration of DMSO, used here as a solvent for FSK, was 0.1% (vol/vol) and thus negligible comparatively to that of 2% (vol/vol) usually required to promote differentiation and polarization of HepaRG cells^2^. As shown in Fig. 1a, control cells, exposed to only 0.1% (vol/vol) DMSO, displayed only a few BC over the whole period of analysis; similarly, cells exposed to FSK for 24 h exhibited little, if any, BC (Fig. 1a). By contrast, HepaRG cells treated by FSK for 48 h to 96 h unambiguously exhibited increased formation of BC (Fig. 1a). In contrast to FSK, 2% (vol/vol) DMSO applied for 72 h failed to enhance BC formation in HepaRG cells (Supplementary Fig. S1), which fully agrees with the fact that a DMSO-treatment for 2 weeks is commonly required to generate differentiated HepaRG cells^1^. Quantification of BC number by image analysis in FSK-treated cell cultures revealed 17.8 ± 2.8, 22.8 ± 2.8 and 25.5 ± 2.4 BC/100 nuclei for cells exposed to 50 µM FSK for 48 h, 72 h or 96 h, respectively, whereas untreated corresponding counterparts showed 3.4 ± 1.0, 4.1 ± 0.7 and 4.8 ± 1.5 BC/100 nuclei (Fig. 1b). The increase of BC number in response to a 72 h-exposure to FSK was concentration-dependent, with an EC_50_ value of 8.3 ± 1.4 µM for FSK (Fig. 1c). Treatment by 50 µM FSK for 72 h was additionally found to significantly enhance BC area size average by 1.6 ± 0.1-fold factor (Fig. 1d). FSK also increased BC number and BC area size average for HepaRG cells plated at low density and continuously exposed to the diterpene for 14 days from cell plating, without addition of 2% (vol/vol) DMSO (Supplementary Fig. S2); BC structures nevertheless appeared in such HepaRG cells seeded at low density only after 8-9 days of culture in the presence of FSK (data not shown). HepaRG cells plated at high density, which display earlier confluency and faster respond to FSK than counterparts seeded at low density, were consequently used for the following experiments.

**Figure 2:**
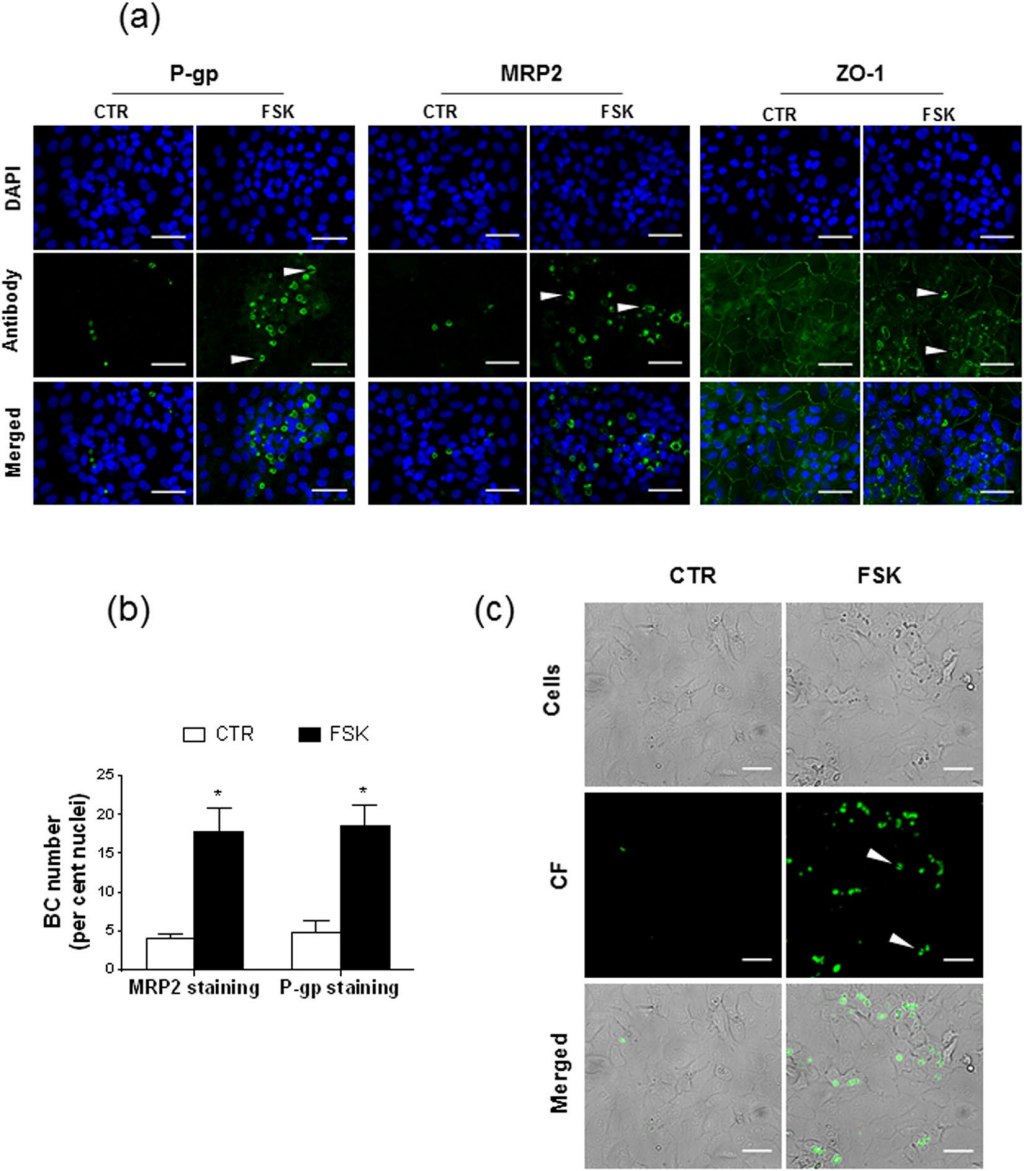
Biliary marker positivity and functionality of FSK-induced BC. (**a**–**c**) HepaRG cells were either exposed to 0.1% (vol/vol) DMSO (control/CTR) or exposed to 50 µM FSK for 72 h. (**a**) The biliary markers P-gp and MRP2 as well as the tight junction marker ZO-1 were immunolocalized, as described in Methods; specific immunolabelling appears as green fluorescence on fluorescence microscopy pictures, whereas DAPI-stained nuclei are blue. BC-associated green fluorescence is indicated by white arrows. (**b**) BC numbers were determined according to P-gp- or MRP2-related immunostaining. (**c**) CF-labeled functional BC were detected through fluorescence microscopy. Data shown are representative (**a**,**c**) or are the means ± SEM (B) of three independent assays. (**a**,**c**) White bar = 50 µm. (**b**) *p < 0.05 when compared to control cells.

FSK effects towards BC marker expression were next studied through immunolocalization. As shown in Fig. 2a, 50 µM FSK stimulated expression of the biliary efflux pumps P-gp and MRP2 at the canalicular pole of HepaRG cells. Quantification of BC formation according to MRP2 or P-gp immunolabelling indicated 17.7 ± 3.2 (MRP2 labelling) and 18.5 ± 2.7 (P-gp labelling) BC/100 nuclei for FSK-treated cells versus 3.9 ± 0.7 (MRP2 labelling) and 4.7 ± 1.6 (P-gp labelling) BC/100 nuclei for control cells (Fig. 2b). Such data concerning BC numbers established from canalicular marker location were similar to those based on the morphological aspect of BC, reported above (Fig. 1b). In addition to MRP2 and P-gp, the tight junction-associated protein ZO-1 was also expressed along BC-like structures in FSK-exposed HepaRG cells, whereas it was more uniformly distributed in control counterparts (Fig. 2a). Functionality of FSK-induced BC was finally studied using the MRP2 fluorescent substrate CF^27^; as shown in Fig. 2c, CF was secreted into BC structures in HepaRG cells exposed to the diterpene.

### Effects of FSK on hepatic marker expression

Expression of various hepatic markers was studied by RT-qPCR in HepaRG cells exposed or not to FSK and expressed as percentage of those found in freshly isolated human hepatocytes (Table 1). When compared to control counterparts, FSK-exposed HepaRG cells showed induced mRNA expressions of several drug detoxifying proteins such as CYP3A4, CYP2C9, UDP-glucuronosyltransferase 1A1 (UGT1A1), NTCP, OATP2B1, MDR1/P-gp and bile salt export pump (BSEP) (Table 1). CYP2B6 mRNA level was also induced by a 2.5-fold factor, without however reaching a statistical significance. FSK additionally increased mRNA expression of the nuclear receptor farnesoid X receptor (FXR) and of various hepatic markers involved directly or indirectly in glucose homeostasis, such as aldolase B, glucose 6 phosphatase (G6P) and tyrosine aminotransferase (TAT), whereas it decreased that of cytokeratin 19, a well-established marker of biliary epithelial cells (Table 1). Nevertheless, when compared to conventionally DMSO-differentiated HepaRG cells, *i.e*., cells seeded at low density and incubated for 14 days in a DMSO-free medium, followed by additional 14 days in the presence of 2% (vol/vol) DMSO, several hepatic markers remained less expressed in FSK-exposed HepaRG cells. It was notably the case for the drug detoxifying proteins CYP2B6, CYP1A2, CYP2C9, CYP2E1, glutathione S-transferase A1 (GSTA1) and organic cation transporter 1 (OCT1), as well as for the nuclear receptors CAR and PXR. Other hepatic markers were similarly expressed (CYP3A4, NTCP, G6P and breast cancer resistance protein (BCRP, also known as ABCG2)) or exhibited increased expression (OATP2B1 and BSEP) in FSK-treated cells when compared to DMSO-treated counterparts (Table 1). It is however noteworthy that mRNA expression of various CYPs, including CYP2B6, CYP1A2, CYP2E1, CYP2D6, as well as those of some transporters like NTCP, OCT1 and OATP1B1, remained much lower in HepaRG cells, even after FSK treatment, than in freshly isolated human hepatocytes, *i.e*., the mRNA levels of these hepatic detoxifying markers in FSK-exposed HepaRG cells represent less than 10% of those found in hepatocytes (Table 1).

**Table 1:** Hepatic marker mRNA expression in FSK-treated HepaRG cells.

Gene	mRNA expression in HepaRG cells (%)^a^		
	Control	FSK	2% DMSO
*Phase I drug metabolizing enzymes*			
CYP3A4	0.74 ± 0.15	57.5 ± 5.9*	62.7 ± 9.7*
CYP2B6	0.83 ± 0.11	2.5 ± 0.2	43.2 ± 7.6#
CYP1A2	0.05 ± 0.01	0.04 ± 0.01	0.61 ± 0.15#
CYP2C9	1.5 ± 0.3	11.0 ± 0.9*	22.8 ± 3.4#
CYP2E1	0.09 ± 0.02	0.07 ± 0.01	2.2 ± 0.1#
CYP2D6	0.49 ± 0.09	0.33 ± 0.08	0.34 ± 0.07
*Phase II drug metabolizing enzymes*			
GSTA1	90.2 ± 22.4	117.2 ± 13.2	165.0 ± 16.3*
UGT1A1	24.5 ± 3.0	64.2 ± 5.5*	107.0 ± 13.5#
*Drug transporters*			
NTCP	3.0 ± 0.3	8.2 ± 0.7*	6.5 ± 0.6*
OCT1	5.4 ± 1.3	5.2 ± 1.1	14.2 ± 2.1#
OATP2B1	22.8 ± 1.5	76.1 ± 11.8*	34.7 ± 3.7¤
OATP1B1	3.4 ± 0.4	5.3 ± 0.8	6.8 ± 0.7
MDR1/P-gp	712.3 ± 69.7	963.4 ± 63.4*	371.2 ± 49.2#
BSEP	9.1 ± 1.2	85.7 ± 8.6*	5.8 ± 0.8¤
BCRP	21.4 ± 2.4	18.3 ± 2.3	18.9 ± 2.8
MRP2	204.8 ± 26.7	296.4 ± 50.5	153.7 ± 33.6¤
MRP3	661.4 ± 99.3	467 ± 96.3	454.4 ± 46.4
*Transcription factors*			
AhR	145.4 ± 18.3	108.7 ± 10.8	95.4 ± 19.1
CAR	2.1 ± 1.0	1.5 ± 1.1	26.9 ± 3.4#
PXR	13.9 ± 2.5	22.2 ± 2.9	42.4 ± 7.4#
FXR	63.3 ± 6.0	103.3 ± 6.2*	76.2 ± 13.3
HNF4α	81.2 ± 16.7	106.8 ± 24.9	68.7 ± 27.5
*Other hepatic markers*			
Albumin	23.0 ± 3.4	22.4 ± 2.4	32.2 ± 6.4
Aldolase B	1.7 ± 0.36	6.1 ± 0.67*	11.2 ± 6.7#
Apolipoprotein B	30.9 ± 3.2	30.9 ± 3.1	20.8 ± 5.3
G6P	0.56 ± 0.11	3.7 ± 0.6*	3.4 ± 0.3*
TAT	0.56 ± 0.16	1.5 ± 0.2*	0.73 ± 0.19
α-fetoprotein	36.1 ± 9.9	44.3 ± 7.3	10.2 ± 2.3¤
CYP7A1	5.5 ± 1.0	9.7 ± 1.2	11.1 ± 2.4*
*Biliary markers*			
Cytokeratin 19	69540 ± 8266	26201 ± 3053*	32394 ± 3746*

^a^Data correspond to mRNA expression in HepaRG cells either exposed to 0.1% (vol/vol) DMSO (control) or exposed to 50 µM FSK for 3 days or to 2% (vol/vol) DMSO for 14 days; they are expressed as percentages of gene expression level found in independent isolated human hepatocytes, arbitrarily set at 100% for each gene, and are the means ± SEM of at least five independent assays. *p < 0.05 when compared to control HepaRG cells; #p < 0.05 when compared to control and FSK-treated HepaRG cells; ¤p < 0.05 when compared to FSK-treated HepaRG cells.

**Figure 3:**
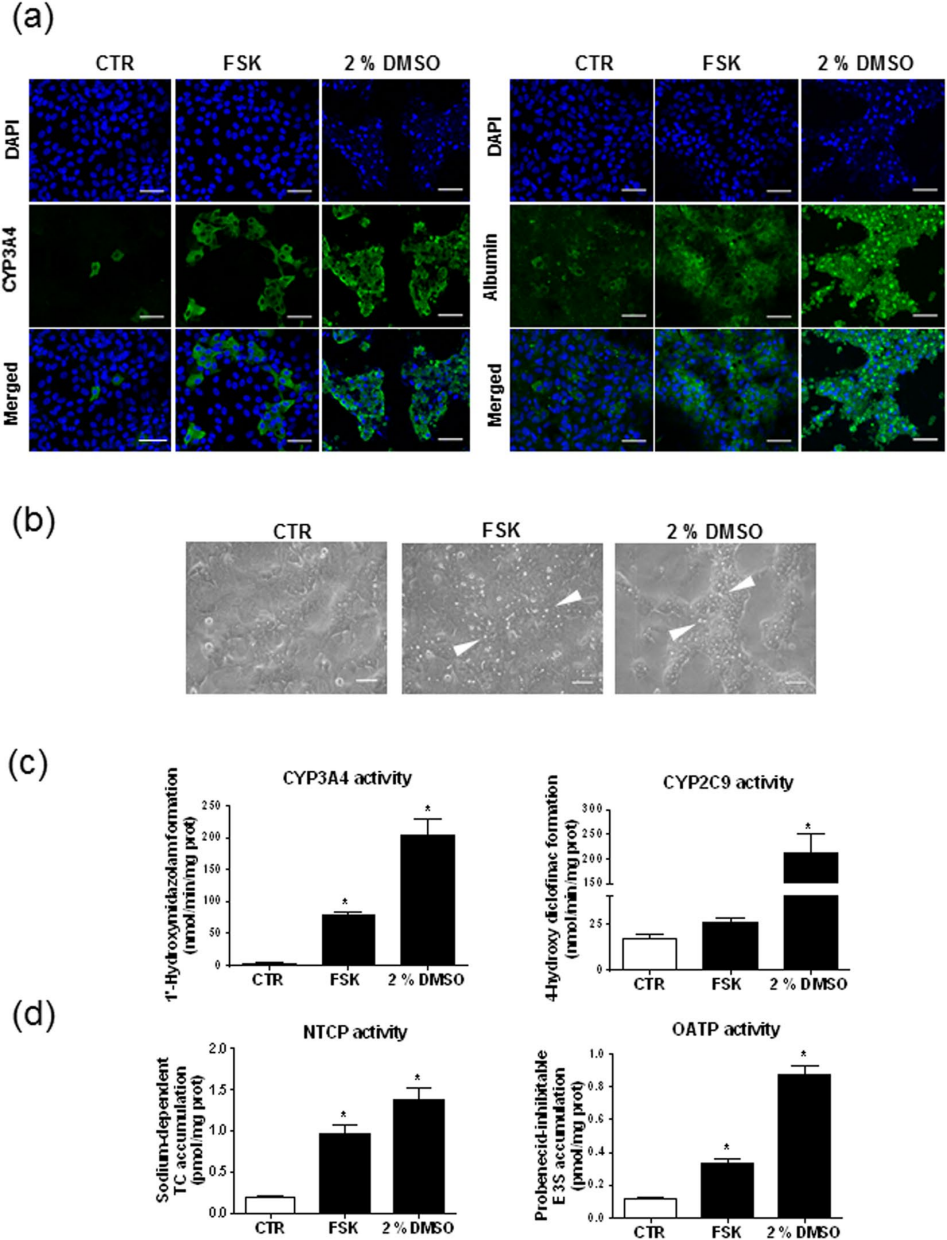
Location and functionality of hepatic markers in FSK-treated HepaRG cells. (**a**–**d**) HepaRG cells were either exposed to 0.1% (vol/vol) DMSO (control/CTR) or treated by 50 µM FSK for 72 h or differentiated with 2% (vol/vol) DMSO for 14 days. (**a**) CYP3A4 and albumin were immunolocalised; specific immunolabelling appears as green fluorescence on fluorescence microscopy pictures, whereas DAPI-stained nuclei are blue. (**b**) Phase-contrast microscopy pictures of cell cultures, with white arrows indicating refractive BC. (**c**) CYP3A4 and CYP2C9 activities were analyzed by LC-MS/MS. (**d**) NTCP and OATP activity were determined through measuring the sodium-dependent uptake of the reference NTCP substrate taurocholate (TC) or the probenecid-inhibitable uptake of the reference OATP substrate E3S. Data shown are representative (**a**,**b**) or are the means ± SEM (**c**,**d**) of three independent assays. (**a**,**b**) White bar = 50 µm. (**c**,**d**) *p < 0.05 when compared to control cells.

The number of CYP3A4-positive cells was next shown to be increased in HepaRG cell cultures exposed to FSK comparatively to untreated counterparts, as indicated by immunolocalization studies (Fig. 3a). Such CYP3A4-positive cells in FSK-treated HepaRG cell cultures however failed to form well-delimited CYP3A4-positive colonies/island of cells, which, by contrast, were observed in cultures exposed to 2% (vol/vol) DMSO. Similarly, immunolocalization studies highlighted albumin-positive cell colonies in DMSO-exposed HepaRG cell cultures, whereas such albumin-positive cells appeared more dispersed in counterparts exposed to FSK (Fig. 3a). In the same way, phase-contrast microscopic analysis highlighted well-defined BC-containing hepatocyte-like colonies surrounded by clear epithelial cells in DMSO-treated cultures, whereas such hepatocytes-like colonies were much less individualized in counterparts exposed to FSK (Fig. 3b).

FSK was additionally shown to increase CYP3A4 activity by a 32.0 ± 7.8-fold factor (Fig. 3c); CYP2C9 activity was also enhanced, but more slightly (by a 1.5 ± 0.1-fold-factor) and without reaching significance. CYP3A4 and CYP2C9 activities remained nevertheless lower than those exhibited by DMSO-treated HepaRG cells (Fig. 3c). FSK also enhanced NTCP and OATP transport activities, without reaching levels found in DMSO-treated cells (Fig. 3d).

### Effects of cAMP analogues on BC formation and hepatic marker expression

**Figure 4:**
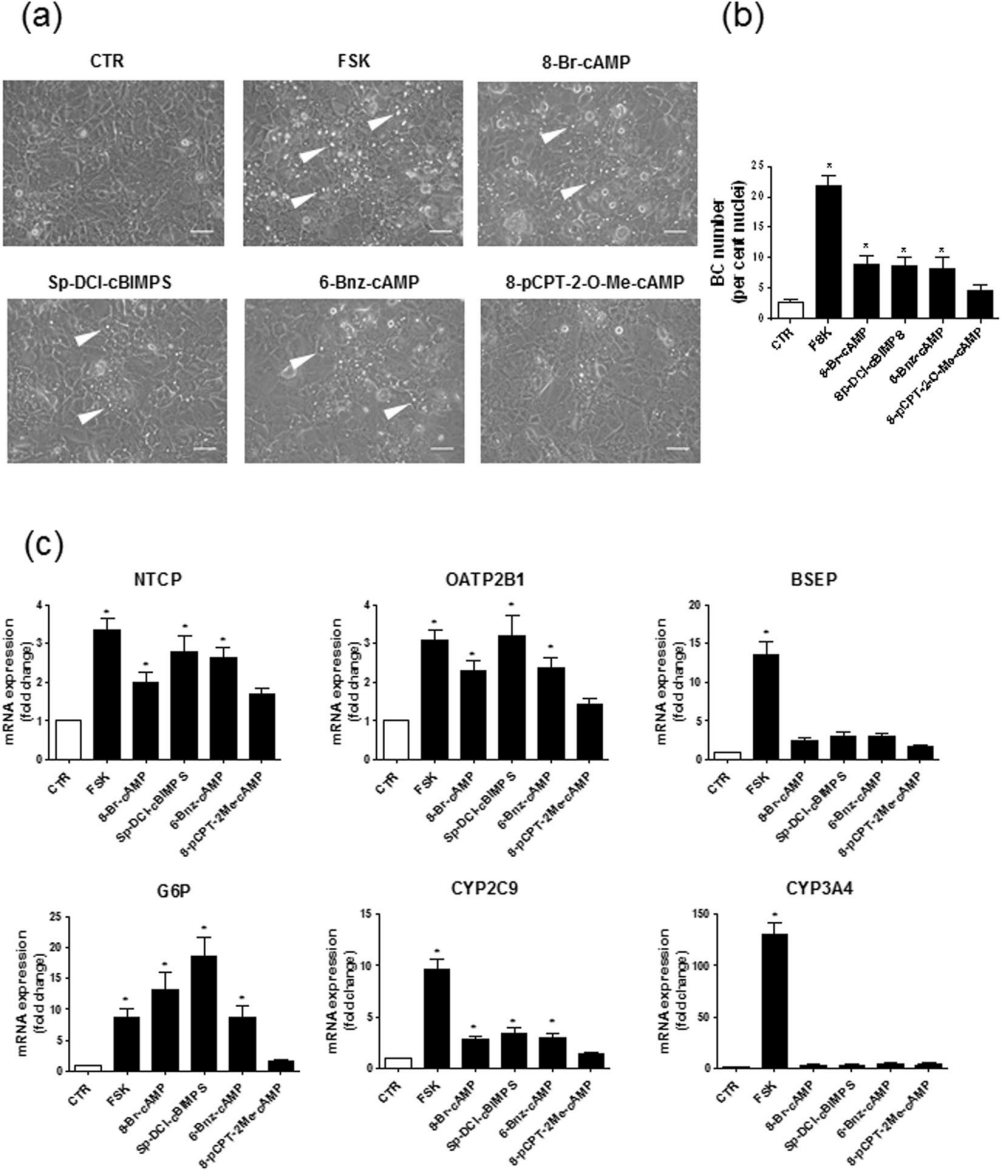
Formation of BC and hepatic marker mRNA expression in HepaRG cells exposed to various cAMP analogs. (**a**–**c**) HepaRG cells were either exposed to 0.1% (vol/vol) DMSO (control/CTR) or treated for 72 h by 50 µM FSK, 500 µM 8-Br-cAMP, 100 µM Sp-5,6-DCl-cBIMPS, 500 µM 6-Bnz-cAMP or 500 µM 8-pCPT-2-O-Me-cAMP. (**a**) Cells were then observed by phase contrast microscopy, allowing to distinguish refractive bright/white BC, indicated by white arrows on phase-contrast microscopic pictures; white bar = 50 µm. (**b**) BC numbers were next quantified by image analysis, as indicated in Methods. (**c**) mRNA expression of various hepatic markers was determined by RT-qPCR; data are expressed as fold change comparatively to untreated control cells. Data shown are representative of 4 independent experiments (**a**) or are the means ± SEM of at least 3 independent assays (**b**,**c**). (**b**,**c**) *p < 0.05 when compared to control cells.

Because FSK is primary known as a cAMP generating agent through adenylate cyclase activation, we studied whether exposure to cAMP analogues may recapitulate FSK effects towards BC formation and hepatic marker expression. We first verified that FSK effectively increased cAMP level in HepaRG cells; as indicated in Supplementary Fig. S3, FSK hugely induced cAMP level in HepaRG cells. We next treated cells by 8-Br-cAMP and Sp-DCl-cBIMPS, activating two major downstream signaling ways of cAMP, *i.e*., protein kinase A (PKA) and exchange protein directly activated by cAMP (Epac), as well as by 6-Bnz-cAMP (thought to act as a rather selective PKA activator)^28^ or by 8-pCPT-2′-O-Me-cAMP (activating only Epac)^29^. As shown in Fig. 4a,b, 8-Br-cAMP, Sp-DCl-cBIMPS and 6-Bnz-cAMP were found to stimulate BC formations in HepaRG cells, but to a lower extent than FSK. The cyclic nucleotide phosphodiesterase inhibitor IBMX, which indirectly enhances intracellular levels of cAMP through inhibiting its degradation^30^, similarly enhanced BC formation in HepaRG cells, but also in a weaker manner than FSK (Supplementary Fig. S4). The cAMP analogues 8-Br-cAMP, Sp-DCl-cBIMPS and 6-Bnz-cAMP were next shown to increase mRNA expression of NTCP, OATP2B1, CYP2C9 and G6P, but they failed to significantly enhance those of CYP3A4 and BSEP (Fig. 4c). By contrast, 8-pCPT-2′-O-Me-cAMP did not favor BC formation (Fig. 4a,b) and did not increase expression of genes regulated by FSK (Fig. 4c).

### Implication of PXR in FSK-mediated BC formation and CYP3A4 regulation

**Figure 5:**
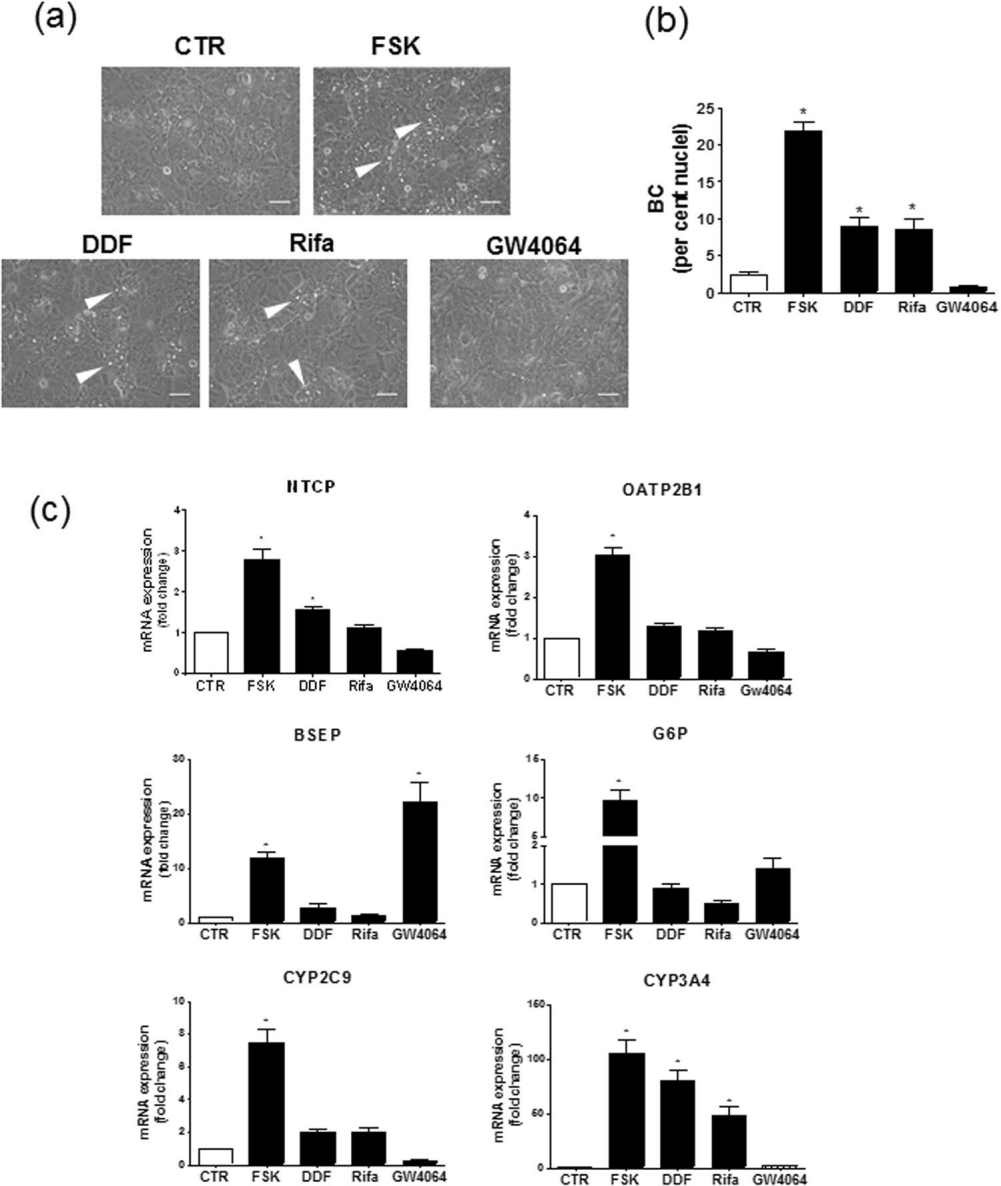
Formation of BC and hepatic marker mRNA expression in HepaRG cells exposed to the PXR agonists DDF or rifampicin or to the FXR agonist GW4064. (**a**–**c**) HepaRG cells were either exposed to 0.1% (vol/vol) DMSO (control/CTR) or treated for 72 h by 50 µM FSK, 50 µM DDF, 50 µM rifampicin (Rifa) or 5 µM GW4064. (**a**) Cells were then observed by phase contrast microscopy, allowing to distinguish refractive bright/white BC, indicated by white arrows on phase-contrast microscopic pictures; white bar = 50 µm. (**b**) BC numbers were next quantified by image analysis, as indicated in Methods. (**c**) mRNA expression of various hepatic markers was determined by RT-qPCR; data are expressed as fold change comparatively to untreated control cells. Data shown are representative of 6 independent experiments (**a**) or are the means ± SEM of at least 6 independent assays (**b**,**c**). (**b**,**c**) *p < 0.05 when compared to control cells.

Besides activating adenylate cyclase, FSK has been shown to exert additional effects, notably to activate the nuclear receptors PXR and FXR^31,32^. To determine whether such nuclear receptors may contribute to FSK effects towards BC formation and gene regulation in HepaRG cell cultures, we treated HepaRG cells by DDF, a structural analogue of FSK activating PXR, but not adenylate cyclase^31^, and by rifampicin and GW4064, reference agonists of PXR and FXR, respectively^33^. Interestingly, DDF and rifampicin, unlike GW4064, were found to trigger BC formation in HepaRG cells, but to a lower extent than FSK (Fig. 5a,b). They also enhanced CYP3A4 mRNA expression, as expected for PXR agonists, but failed to increase, or only weakly induced, other hepatic markers such as NTCP, OATP2B1, G6P and BSEP (Fig. 5c). GW4064 was found to induce mRNA expression of the reference FXR target BSEP (Fig. 5c), confirming thus the efficiency of this FXR agonist in our cell cultures.

**Figure 6:**
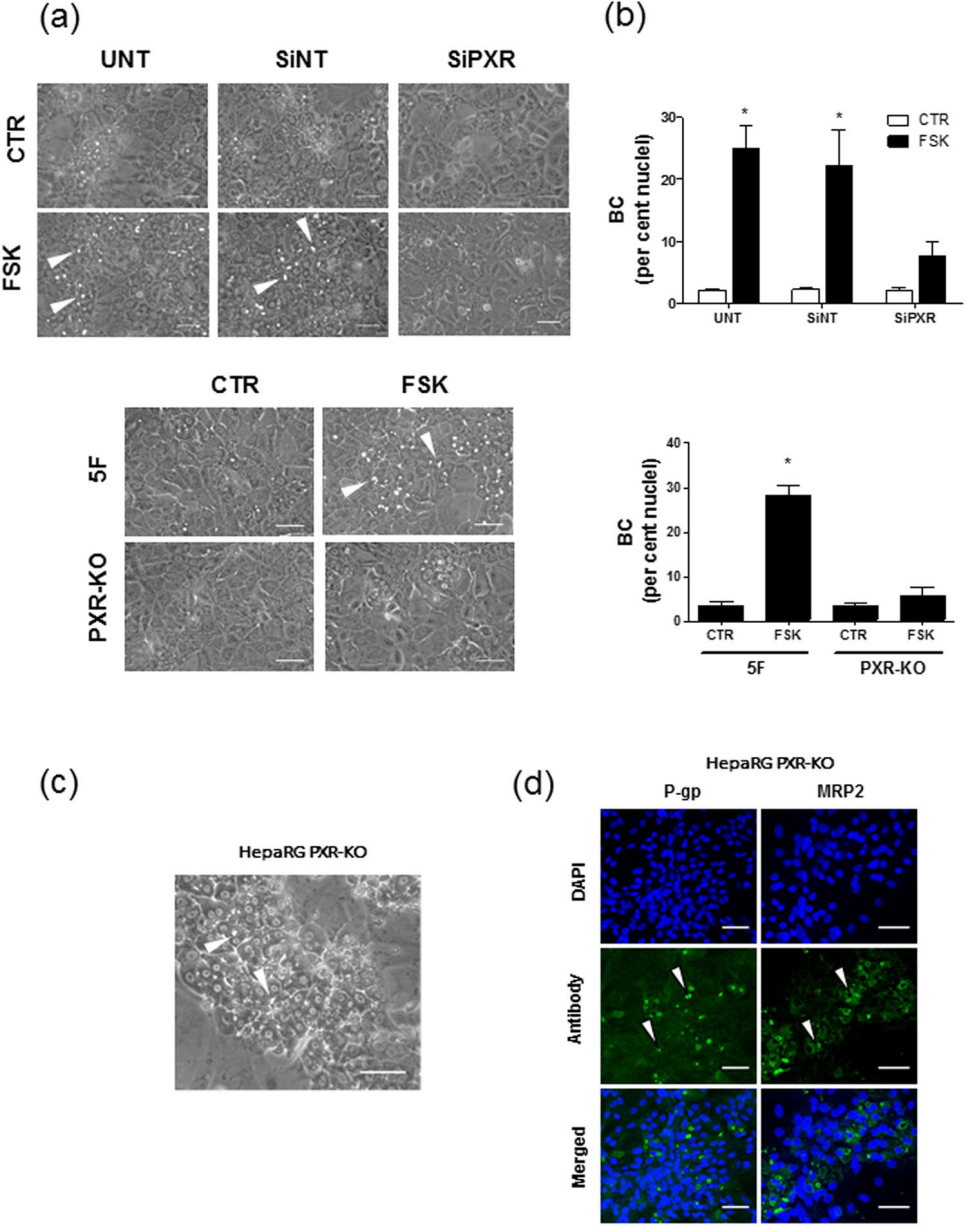
Effects of PXR silencing on FSK-induced formation of BC. (**a**,**b**) Untransfected (UNT) and SiNT- or SiPXR-transfected HepaRG cells as well as PXR-KO and control F5 HepaRG clone cells were either exposed to 0.1% (vol/vol) DMSO (control/CTR) or treated by 50 µM FSK for 72 h. (**a**) Cells were then observed by phase contrast microscopy, allowing to distinguish refractive bright/white BC, indicated by white arrows on phase-contrast microscopic pictures. (**b**) BC numbers were next quantified by image analysis, as indicated in Methods; data shown are the means ± SEM of at least three independent assays. *p < 0.05 when compared to control cells not exposed to FSK (**c**,**d**) HepaRG PXR-KO cells were cultured for 18 days. (**c**) Cells were then observed by phase contrast microscopy, allowing to distinguish refractive bright/white BC, indicated by white arrows on phase-contrast microscopic pictures. (**d**) The biliary markers P-gp and MRP2 were immunolocalized, as described in Methods; specific immunolabelling appears as green fluorescence on fluorescence microscopy pictures, whereas DAPI-stained nuclei are blue; BC-associated green fluorescence is indicated by white arrows; the data shown are representative of three independent assays. (**a**,**c**,**d**) White bar = 50 µm.

To further analyze the possible implication of PXR in FSK polarizing activity, we studied the effect of siRNA-mediated PXR silencing on FSK effects in HepaRG cells. The efficiency of the siRNA-mediated approach was demonstrated by its capacity to decrease PXR protein expression (Supplementary Fig. S5a) and to hinder FSK-mediated CYP3A4 up-regulation (Supplementary Fig. S5b) in HepaRG cells. SiRNA-mediated depletion of PXR was found to markedly reduce BC formation due to FSK treatment, without however fully abolishing it (Fig. 6a,b). It however failed to alter G6P up-regulation due to FSK (Supplementary Fig. S5b). PXR role in FSK-mediated BC formation was further studied using PXR-KO HepaRG cells and corresponding control F5 HepaRG cells^34^. PXR-KO cells failed to express PXR as demonstrated by Western-blotting (Supplementary Fig. S5a); they did not exhibit FSK-mediated up-regulation of CYP3A4 mRNA, whereas that of G6P was preserved (Supplementary Fig. S5b). FSK-mediated BC formation was found to be nearly fully abrogated in PXR-KO HepaRG cells, but occurred in control F5 cells (Fig. 6a,b). When cultured for 18 days at confluency in basal conditions (absence of FSK and of DMSO), PXR-KO HepaRG cells were however able to spontaneously develop BC structures (Fig. 6c), which expressed the canalicular markers MRP2 and P-gp (Fig. 6d). Such data suggest that PXR may be required for BC formation triggered by FSK, but not by confluency time, in HepaRG cells.

### Recapitulation of FSK effects by the combination cAMP analog/rifampicin

**Figure 7:**
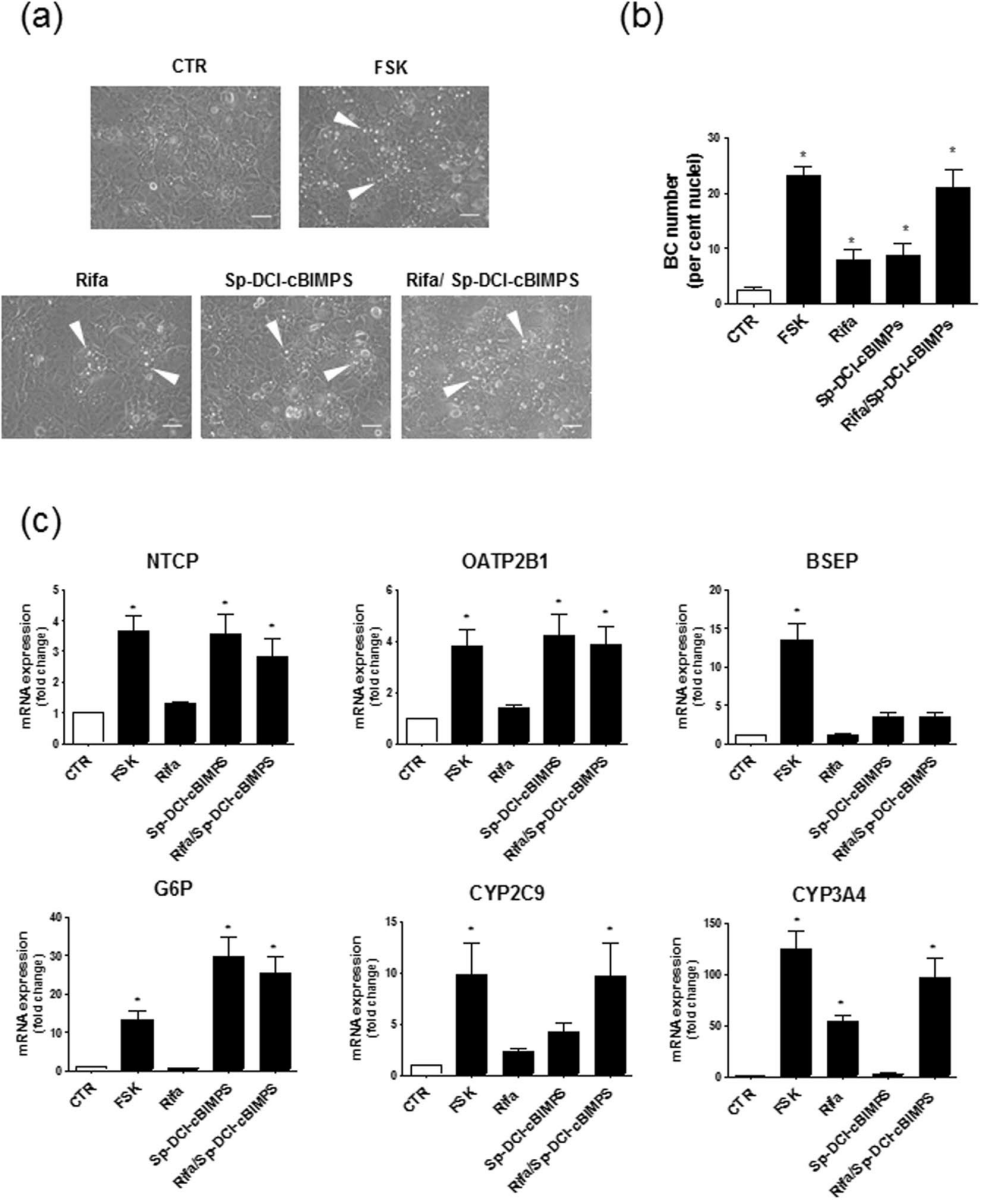
Effects of cAMP/rifampicin on BC formation and hepatic marker expression in HepaRG cells. (**a**–**c**) HepaRG cells were either exposed to 0.1% (vol/vol) DMSO (control/CTR) or treated for 72 h by 50 µM FSK, 50 µM rifampicin (Rifa), 100 µM Sp-5,6-DCl-cBIMPS or the combination 50 µM rifampicin/100 µM Sp-5,6-DCl-cBIMPS. (**a**) Cells were then observed by phase contrast microscopy, allowing to distinguish refractive bright/white BC, indicated by white arrows on phase-contrast microscopic pictures; white bar = 50 µm. (**b**) BC numbers were next quantified by image analysis, as indicated in Methods. (**c**) mRNA expression of various hepatic markers was determined by RT-qPCR; data are expressed as fold change comparatively to untreated control cells. Data shown are representative of 4 independent experiments (**a**) or are the means ± SEM of at least 3 independent assays (**b**,**c**). (**b**,**c**) *p < 0.05 when compared to control cells.

Because both PXR activation by rifampicin and cAMP treatment partially induced BC formation when compared to that triggered by FSK, we wonder whether the combination cAMP/rifampicin may recapitulate FSK effects towards BC formation in HepaRG cells. As shown in Fig. 7a,b, co-treatment by rifampicin and Sp-DCl-cBIMPS, but not by rifampicin/8-pCPT-2′-O-Me-cAMP (data not shown), was found to enhance BC formation in a similar manner than FSK. The combination Sp-DCl-cBIMPS/rifampicin also enhanced mRNA expression of NTCP, OATP2B1, CYP3A4, CYP2C9 and G6P, as did it FSK (Fig. 7c); only BSEP mRNA level was not up-regulated by Sp-DCl-cBIMPS/rifampicin co-treatment (Fig. 7c).

## Discussion

The data reported in the present study indicated that a relative short-term (72 h) treatment by FSK is able to induce functional polarization of human hepatoma HepaRG cells, without addition of the differentiating agent DMSO, commonly used for getting polarized HepaRG cells^1,3,4^. Indeed, FSK treatment triggers the appearance of BC structures, expressing biliary markers like the efflux pumps P-gp and MRP2. Moreover, these BC appear to be functional, as demonstrated by the secretion of the MRP2 substrate CF into their lumen. Such data therefore confirm the fact that FSK can act as a polarizing agent for hepatocytes, as already described for rat hepatocytes and human HepG2 cells^18,19^. However, for HepG2 cells, in contrast to HepaRG cells, BC structures are likely difficult to detect by phase-contrast microscopy and mislocalization of apical markers may occur^35^.

In addition to stimulate polarization, FSK unambiguously increased mRNA expression of hepatic markers in HepaRG cells, notably those of the drug metabolizing enzymes CYP3A4 and UGT1A1, the drug transporters NTCP, OATP2B1 and BSEP and the glucose metabolism enzymes aldolase B and G6P. FSK concomitantly robustly induced CYP3A4 activity and NTCP and OATP activity. In contrast, expression of the biliary marker cytokeratin 19 was decreased. Such data suggest that FSK may act as a global differentiating agent towards HepaRG cells, through favoring the acquisition of key hallmarks of the differentiated hepatocyte, like a polarized status and the expression of hepatic enzymes involved in drug or carbohydrate metabolism. Such differentiation features are similarly promoted by treating HepaRG cells with DMSO^1,6^. In this context, it is however noteworthy that FSK failed to recapitulate all the differentiating effects of DMSO. This conclusion is supported by the following points: (i) FSK, unlike DMSO, did not augment mRNA expression of some major hepatic CYPs, such as CYP1A2 and CYP2E1, whereas it only marginally enhanced that of CYP2B6, (ii) FSK-exposed HepaRG cells, although exhibiting notable increase of CYP2C9 mRNA levels, exhibited much lower CYP2C9 activity than DMSO-treated counterparts, (iii) expression of the drug sensing receptor CAR, known to stimulate the differentiation and maturation of hepatic-like cells^12^, including those of HepaRG cells^13^, remained low in FSK-treated HepaRG cell cultures, and (iv) FSK-exposed cells failed to display the well-delimited islands of CYP3A4/albumin-positive hepatocyte-like cells, observed in DMSO-treated counterparts. The profile of hepatic differentiation in FSK-treated HepaRG cells is therefore only partly identical to that found in DMSO-treated counterparts. The fact that mRNA expression of the canalicular marker BSEP was markedly induced by FSK, but not by DMSO, supports this conclusion.

**Figure 8:**
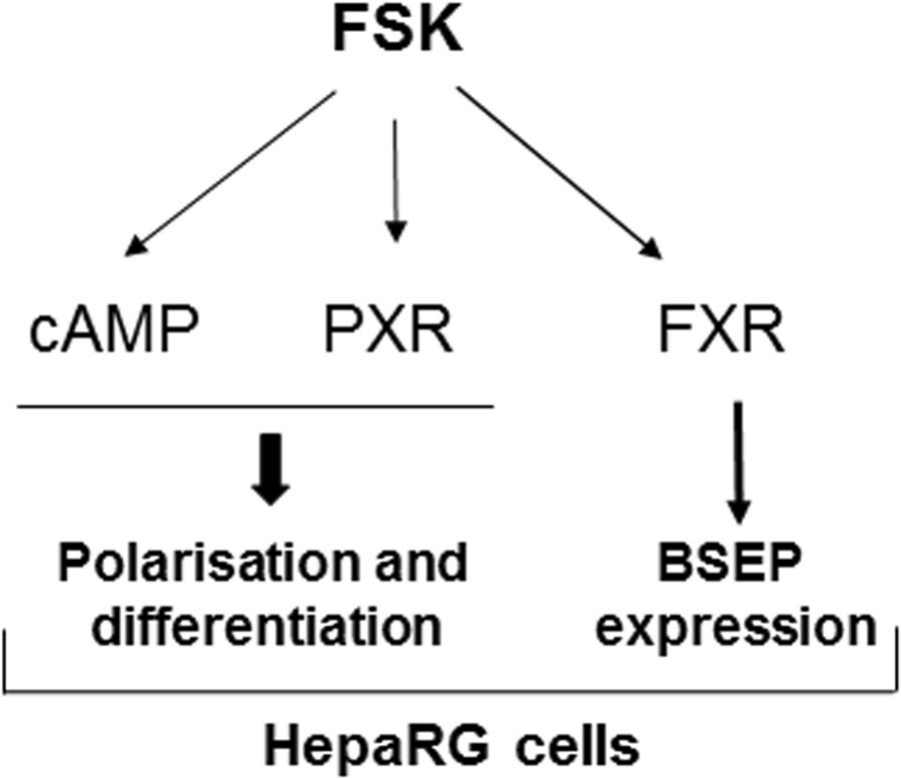
Schematic representation of the signaling ways that may contribute to the FSK effects in HepaRG cells.

FSK is primarily known to basically stimulate the production of cAMP through adenylate cyclase activation^14,15^. FSK has more recently been shown to activate other targets such as the receptors PXR and FXR^31,32^. Interestingly, this multifaceted activity of FSK is most likely involved in its polarizing and differentiating effects towards HepaRG cells. Indeed, various cAMP analogs, like FSK, were shown to stimulate expression of some hepatic markers, such as NTCP, OATP2B1 and G6P, in HepaRG cells. They additionally partly favored the development of BC. The phosphodiesterase inhibitor IBMX, known to indirectly raised intracellular cAMP levels, also partly stimulated BC formation in HepaRG cells, thus confirming the link between cAMP levels and HepaRG cell polarization. In the same way, PXR agonists, like rifampicin and DDF, a FSK analog devoid of adenylate cyclase activation property^31^, partially promoted the formation of BC; they additionally enhanced the expression of CYP3A4, well-known to be a reference PXR target^36^. The involvement of PXR in polarizing effects of FSK is also supported by the fact that FSK-mediated BC formation and CYP3A4 up-regulation in HepaRG cells were impaired by PXR depletion, achieved transiently through siRNA-transfection or stably through the use of PXR-KO HepaRG cells. The FXR agonist GW4064 was additionally shown to induce BSEP expression, like FSK, without however stimulating BC development. Polarizing and differentiating effects of FSK towards HepaRG cells may therefore reflect its combined effects on cAMP level and PXR- and FXR-signaling ways (See Fig. 8 for a schematic representation of putative signaling ways contributing to FSK effects in HepaRG cells). This hypothesis is supported by the fact that co-treatment by the cAMP analog Sp-DCl-cBIMPS and rifampicin fully reproduced the polarizing and differentiating effects of FSK, except for the up-regulation of BSEP, most likely due to FSK-mediated FXR activation.

The exact signaling ways by which cAMP and PXR promote BC formation in HepaRG cells remain to be determined. The role of main cAMP-downstream effectors, Epac and PKA, were analyzed by using specific cAMP analogs. An implication of Epac seems unlikely, because the Epac-activating cAMP analog 8-pCPT-2′-O-Me-cAMP induced neither BC formation nor hepatic marker expression in HepaRG cells, in contrast to the PKA-activating analog 6-Bnz-cAMP, which is inactive towards Epac^28^. A contribution of PKA may have consequently to be considered, without, however, formally discarding non-canonical cAMP-related ways, described in various cell lines^37–40^ and implicating diverse emergent cAMP effectors proteins, such as cyclic-nucleotide gated channels^41^, Popeye domain containing proteins^42^ or the cAMP sensor Rapgef2^43^. Further studies are consequently required to identify the exact nature of the cAMP effector protein that mediates cAMP-related polarizing/differentiating effects of FSK in HepaRG cells. With respect to PXR, it is most likely involved in FSK-mediated up-regulation of CYP3A4 in HepaRG cells, through transcriptionally activating this reference PXR target gene^36^. The contribution of PXR to polarizing effects of FSK seems to be more complex to apprehend. Indeed, if PXR appears to be at least partly required for FSK-mediated formation of BC in HepaRG cells, its absence in PXR-KO HepaRG cells did not preclude the polarization of these cells with confluency time, indicating that BC formation may arise in the absence of PXR. The fact that alteration of BC has not been reported in the liver of Pxr-KO mouse supports this conclusion^44,45^. In the same way, the effects of PXR towards hepatic differentiation of hESC are complex; thus, microbial-derived lithocholic acid and vitamin K2 have been reported to drive PXR-dependent metabolic maturation of hESC-derived hepatocytes^46^, whereas, by contrast, stable overexpression of PXR in hepatic-induced hESCs failed to enhance expression of hepatic phenotype marker^12^. Additional studies are therefore welcome to precise how activation of PXR may contribute to BC formation in FSK-exposed HepaRG cells and, beyond, to define the exact role of PXR in hepatic polarization and differentiation processes. Nevertheless, our data, through supporting a function for PXR in BC formation, reinforce the conclusion that PXR exerts pleiotropic roles in cellular metabolism and physiology, apart from that initially devoted to drug detoxification pathway regulation^47–49^.

The HepaRG cell line is now well-recognized as an original and convenient *in vitro* model for pharmacological and toxicological studies, acting as a surrogate for primary cultures of human hepatocytes^4–6^. The use of HepaRG cells may however be hampered by the necessity of adding the non-physiological and potentially toxic agent DMSO in culture medium during a relative long culture time (14 days) for getting differentiated cells. In this context, the alternative use of FSK-treated HepaRG cells may be interesting to consider as it permits to discard DMSO and to obtain polarized cells after a short-time treatment (3 days), if done with high density-plated cells. Moreover, these FSK-treated HepaRG cells exhibit various hepatic differentiated features, including expression of CYP3A4 and drug transporters like NTCP, OATP2B1, MRP2 and BSEP, even if other hepatic markers like CYP1A2, CYP2E1 and CAR remain at levels much lower than those found in DMSO-treated counterparts, as already discussed above. Additional works are needed to determine the potential relevance of FSK-treated HepaRG cells as an *in vitro* model for pharmacological-toxicological studies and also to improve it with respect to expression of some hepatic markers.

In summary, FSK was shown to polarize and differentiate human hepatoma HepaRG cells, without the addition of DMSO. This most likely occurs through mobilization of the multifaceted activities of the diterpene, *i.e*., generation of cAMP and activation of the nuclear receptors PXR and FXR. Such effects of FSK may help to ameliorate the use of HepaRG cells as surrogates for human hepatocytes in *in vitro* hepatic studies and also suggest a previously-unrecognized putative role for PXR in hepatocyte polarization.

## Electronic supplementary material

Supplementary information

## References

[1] Gripon P (2002). Infection of a human hepatoma cell line by hepatitis B virus. Proc Natl Acad Sci USA.

[2] Aninat C (2006). Expression of cytochromes P450, conjugating enzymes and nuclear receptors in human hepatoma HepaRG cells. Drug Metab Dispos.

[3] Le Vee M, Noel G, Jouan E, Stieger B, Fardel O (2013). Polarized expression of drug transporters in differentiated human hepatoma HepaRG cells. Toxicol In Vitro.

[4] Guillouzo A (2007). The human hepatoma HepaRG cells: a highly differentiated model for studies of liver metabolism and toxicity of xenobiotics. Chem Biol Interact.

[5] Andersson TB, Kanebratt KP, Kenna JG (2012). The HepaRG cell line: a unique *in vitro* tool for understanding drug metabolism and toxicology in human. Expert Opin Drug Metab Toxicol.

[6] Antherieu S, Chesne C, Li R, Guguen-Guillouzo C, Guillouzo A (2012). Optimization of the HepaRG cell model for drug metabolism and toxicity studies. Toxicol In Vitro.

[7] Nikolaou Nikolaos, Green Charlotte J., Gunn Pippa J., Hodson Leanne, Tomlinson Jeremy W. (2016). Optimizing human hepatocyte models for metabolic phenotype and function: effects of treatment with dimethyl sulfoxide (DMSO). Physiological Reports.

[8] Hoekstra R (2011). The HepaRG cell line is suitable for bioartificial liver application. Int J Biochem Cell Biol.

[9] Nibourg GA (2012). Liver progenitor cell line HepaRG differentiated in a bioartificial liver effectively supplies liver support to rats with acute liver failure. PLoS One.

[10] Rebelo SP (2015). HepaRG microencapsulated spheroids in DMSO-free culture: novel culturing approaches for enhanced xenobiotic and biosynthetic metabolism. Arch Toxicol.

[11] van Wenum M (2018). Oxygen drives hepatocyte differentiation and phenotype stability in liver cell lines. J Cell Commun Signal.

[12] Chen F, Zamule SM, Coslo DM, Chen T, Omiecinski CJ (2013). The human constitutive androstane receptor promotes the differentiation and maturation of hepatic-like cells. Dev Biol.

[13] van der Mark VA (2017). Stable overexpression of the constitutive androstane receptor reduces the requirement for culture with dimethyl sulfoxide for high drug metabolism in HepaRG cells. Drug Metab Dispos.

[14] Seamon KB, Padgett W, Daly JW (1981). Forskolin: unique diterpene activator of adenylate cyclase in membranes and in intact cells. Proc Natl Acad Sci USA.

[15] Sapio L (2017). The Natural cAMP elevating compound forskolin in cancer therapy: Is it time?. J Cell Physiol.

[16] Ranta T, Knecht M, Darbon JM, Baukal AJ, Catt KJ (1984). Induction of granulosa cell differentiation by forskolin: stimulation of adenosine 3′,5′-monophosphate production, progesterone synthesis, and luteinizing hormone receptor expression. Endocrinology.

[17] Wice B, Menton D, Geuze H, Schwartz AL (1990). Modulators of cyclic AMP metabolism induce syncytiotrophoblast formation *in vitro*. Exp Cell Res.

[18] Fu D, Wakabayashi Y, Ido Y, Lippincott-Schwartz J, Arias IM (2010). Regulation of bile canalicular network formation and maintenance by AMP-activated protein kinase and LKB1. J Cell Sci.

[19] Zegers MM, Hoekstra D (1997). Sphingolipid transport to the apical plasma membrane domain in human hepatoma cells is controlled by PKC and PKA activity: a correlation with cell polarity in HepG2 cells. J Cell Biol.

[20] Ogawa S (2013). Three-dimensional culture and cAMP signaling promote the maturation of human pluripotent stem cell-derived hepatocytes. Development.

[21] Burbank MG (2016). Early alterations of bile canaliculi dynamics and the Rho kinase/myosin light chain kinase pathway are characteristics of drug-induced intrahepatic cholestasis. Drug metabolism and disposition: the biological fate of chemicals.

[22] Schneider CA, Rasband WS, Eliceiri KW (2012). NIH Image to ImageJ: 25 years of image analysis. Nat Methods.

[23] Le Vee M, Lecureur V, Stieger B, Fardel O (2009). Regulation of drug transporter expression in human hepatocytes exposed to the proinflammatory cytokines tumor necrosis factor-alpha or interleukin-6. Drug Metab Dispos.

[24] Lecureur V (2005). ERK-dependent induction of TNFalpha expression by the environmental contaminant benzo(a)pyrene in primary human macrophages. FEBS Lett.

[25] Jigorel E, Le Vee M, Boursier-Neyret C, Bertrand M, Fardel O (2005). Functional expression of sinusoidal drug transporters in primary human and rat hepatocytes. Drug Metab Dispos.

[26] Bradford MM (1976). A rapid and sensitive method for the quantitation of microgram quantities of protein utilizing the principle of protein-dye binding. Anal Biochem.

[27] Payen L, Courtois A, Campion JP, Guillouzo A, Fardel O (2000). Characterization and inhibition by a wide range of xenobiotics of organic anion excretion by primary human hepatocytes. Biochem Pharmacol.

[28] Poppe H (2008). Cyclic nucleotide analogs as probes of signaling pathways. Nat Methods.

[29] Enserink JM (2002). A novel Epac-specific cAMP analogue demonstrates independent regulation of Rap1 and ERK. Nat Cell Biol.

[30] Essayan DM (2001). Cyclic nucleotide phosphodiesterases. J Allergy Clin Immunol.

[31] Ding X, Staudinger JL (2005). Induction of drug metabolism by forskolin: the role of the pregnane X receptor and the protein kinase a signal transduction pathway. J Pharmacol Exp Ther.

[32] Howard WR, Pospisil JA, Njolito E, Noonan DJ (2000). Catabolites of cholesterol synthesis pathways and forskolin as activators of the farnesoid X-activated nuclear receptor. Toxicol Appl Pharmacol.

[33] Jonker JW, Liddle C, Downes M (2012). FXR and PXR: potential therapeutic targets in cholestasis. J Steroid Biochem Mol Biol.

[34] Williamson B, Lorbeer M, Mitchell MD, Brayman TG, Riley RJ (2016). Evaluation of a novel PXR-knockout in HepaRG^TM^ cells. Pharmacol Res Perspect.

[35] Decaens C, Durand M, Grosse B, Cassio D (2008). Which *in vitro* models could be best used to study hepatocyte polarity?. Biol Cell.

[36] Lehmann JM (1998). The human orphan nuclear receptor PXR is activated by compounds that regulate CYP3A4 gene expression and cause drug interactions. J Clin Invest.

[37] Iacovelli L (2001). Thyrotropin activates mitogen-activated protein kinase pathway in FRTL-5 by a cAMP-dependent protein kinase A-independent mechanism. Mol Pharmacol.

[38] Ivins JK, Parry MK, Long DA (2004). A novel cAMP-dependent pathway activates neuronal integrin function in retinal neurons. J Neurosci.

[39] Gambaryan S (2006). Regulation of aldosterone production from zona glomerulosa cells by ANG II and cAMP: evidence for PKA-independent activation of CaMK by cAMP. Am J Physiol Endocrinol Metab.

[40] Emery AC, Eiden LE (2012). Signaling through the neuropeptide GPCR PAC_1_ induces neuritogenesis via a single linear cAMP- and ERK-dependent pathway using a novel cAMP sensor. FASEB J.

[41] Biel, M. & Michalakis, S. Cyclic nucleotide-gated channels. *Handb Exp Pharmacol*, 111–136, 10.1007/978-3-540-68964-5_7 (2009). 10.1007/978-3-540-68964-5_7 19089328

[42] Schindler RF, Brand T (2016). The Popeye domain containing protein family–A novel class of cAMP effectors with important functions in multiple tissues. Prog Biophys Mol Biol.

[43] Emery AC, Eiden MV, Eiden LE (2014). Separate cyclic AMP sensors for neuritogenesis, growth arrest, and survival of neuroendocrine cells. J Biol Chem.

[44] Xie W (2000). Humanized xenobiotic response in mice expressing nuclear receptor SXR. Nature.

[45] Staudinger JL (2001). The nuclear receptor PXR is a lithocholic acid sensor that protects against liver toxicity. Proc Natl Acad Sci USA.

[46] Avior Y (2015). Microbial-derived lithocholic acid and vitamin K_2_ drive the metabolic maturation of pluripotent stem cells-derived and fetal hepatocytes. Hepatology.

[47] Hakkola J, Rysa J, Hukkanen J (2016). Regulation of hepatic energy metabolism by the nuclear receptor PXR. Biochim Biophys Acta.

[48] Oladimeji PO, Chen T (2018). PXR: More than just a master xenobiotic receptor. Mol Pharmacol.

[49] Moreau A, Vilarem MJ, Maurel P, Pascussi JM (2008). Xenoreceptors CAR and PXR activation and consequences on lipid metabolism, glucose homeostasis, and inflammatory response. Mol Pharm.

