# Supplementary material for: Functional polarization of human hepatoma HepaRG cells in response to forskolin
Source: Sci Rep. 2018 Oct 31;8:16115. doi: 10.1038/s41598-018-34421-8 (PMC6208432; doi:10.1038/s41598-018-34421-8)
Supplement: Supplementary file 1 — Supplementary information [file 41598_2018_34421_MOESM1_ESM.pdf]

Supplementary information

Functional polarization of human hepatoma HepaRG cells in response to forskolin

Abdullah Mayati[1], Amélie Moreau[2], Marc Le Vée[1], Arnaud Bruyère[1], Elodie Jouan[1], Claire Denizot[2], Yannick Parmentier[2], Olivier Fardel[1,3]

[1]Univ Rennes, Inserm, EHESP, Irset (Institut de recherche en santé, environnement et travail) - UMR_S 1085, F-35000, Rennes, France

[2]Centre de Recherche en Pharmacocinétique, Technologie Servier, F-45000 Orléans, France

[3]Pôle Biologie, Centre Hospitalier Universitaire, F-35033 Rennes, France

Correspondence and requests for materials should be addressed to O.F. (email: olivier.fardel@univ-rennes1.fr)

Supplementary Table S1: Gene primers used for qPCR assays

Gene	Forward Primer (5′-3′)	Reverse Primer (5′-3′)
**CYP3A4**	CTTCATCCAATGGACTGCATAAAT	TCCCAAGTATAACACTCTACACAGACAA
**CYP2B6**	TTCCTACTGCTTCCGTCTATCAAA	GTGCAGAATCCCACAGCTCA
**CYP1A2**	TGGAGACCTTCCGACACTCCT	CGTTGTGTCCCTTGTTGTGC
**CYP2C9**	GGTGTGATTGGCAGAAACC	GTGCCCTTGGGGAATGAGAT
**CYP2D6**	CAGAGATGGAGAAGGCCAAG	CCCTATCACGTCGTCCATCT
**GSTA1**	TATGATGGCTCGAAGGCTCT	GTCTTGTCCATGGCTCTTTAAG
**UGTA1**	TGACGCCTCGTTGTACATCAG	CCTCCCTTTGGAATGGCAC
**NTCP**	GGGACATGAACCTCAGCATT	CGTTTGGATTTGAGGACGAT
**OCT1**	TAATGGACCACATCGCTCAA	AGCCCCTGATAGAGCACAGA
**OATP2B1**	TGATTGGCTATGGGGCTATC	CATATCCTCAGGGCTGGTGT
**OATP1B1**	GCCCAAGAGATGATGCTTGT	ATTGAGTGGAAACCCAGTGC
**MDR1/P-gp**	GCCAAAGCCAAAATATCAGC	TTCCAATGTGTTCGGCATTA
**BSEP**	TGATCCTGATCAAGGGAAGG	TGGTTCCTGGGAAACAATTC
**BCRP**	TGCAACATGTACTGGCGAAGA	TCTTCCACAAGCCCCAGG
**MRP2**	TGAGCAAGTTTGAAACGCACAT	AGCTCTTCTCCTGCCGTCTCT
**MRP3**	GTCCGCAGAATGGACTTGAT	TCACCACTTGGGGATCATTT
**AhR**	CTTCCAAGCGGCATAGAGAC	AG TTATCCTGGCCTCCGTTT
**CAR**	TGATCAGCTGCAAGAGGAGA	AGGCCTAGCAACTTCGCATA
**PXR**	CCAGGACATACACCCCTTTG	CTACCTGTGATGCCGAACAA
**FXR**	GGAGGATCAAAGGGGATGA	CAGTTGCCCCCGTTTTTAC
**HNF4α**	AACCTGTTGCAGGAGATGC	CGTTGGTTCCCATATGTTCC
**α-fetoprotein**	TGCAGCCAAAGTGAAGAGGGAAGA	CATAGCGAGCAGCCCAAAGAAG
**CYP7A1**	AGACACACCTCGTGGTCCTC	TTTCATTGCTTCTGGGTTCC
**18S**	CGCCGCTAGAGGTGAAATTC	TTGGCAAATGCTTTCGCTC

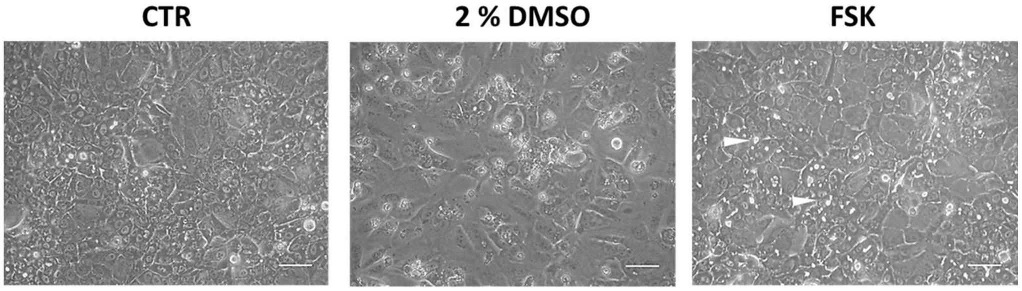

Supplementary Fig. S1. Differential effects of 72 h-treatment by FSK or 2 % (vol/vol) DMSO on formation of refractive BC-like structures in HepaRG cells

HepaRG cells plated at high density were either exposed to 0.1 % (vol/vol) (Control/CTR) or 2 % (vol/vol) DMSO or treated by 50 µM FSK for 72 h. Cells were then observed by phase contrast microscopy, allowing to distinguish refractive bright/white BC, indicated by white arrows on phase-contrast microscopic pictures of FSK-treated HepaRG cells; white bar = 50 µm. Data shown are representative of 3 independent assays.

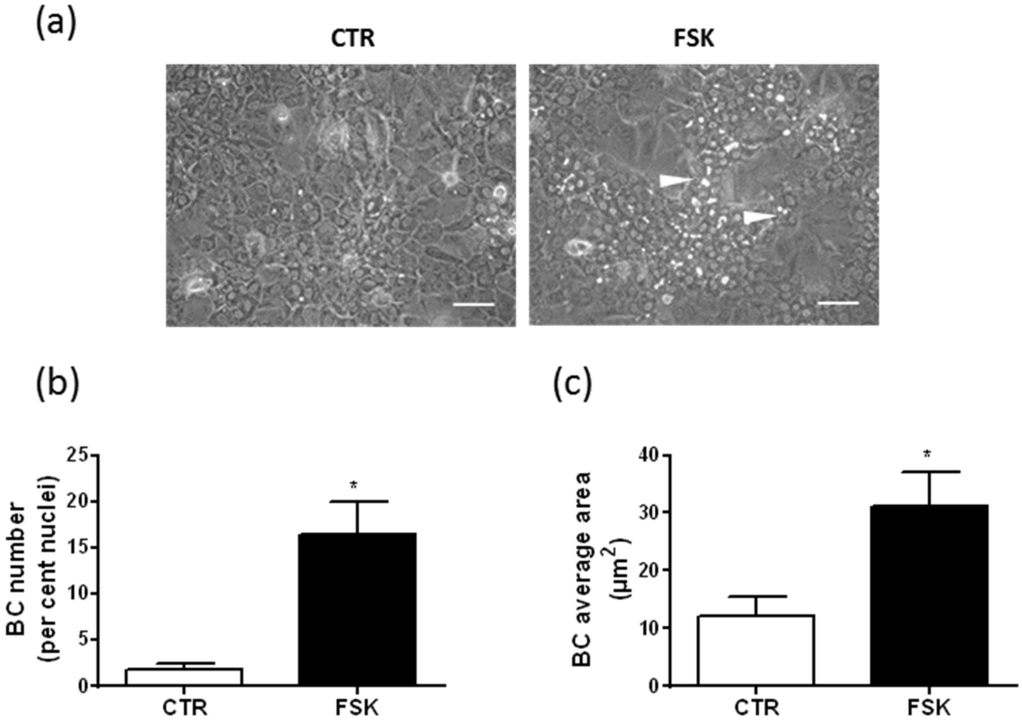

Supplementary Fig. S2. Formation of refractive BC-like structures in low density-plated HepaRG cells exposed to FSK.

(a-c) HepaRG cells plated at low density were either exposed to 0.1 % (vol/vol) DMSO (control/CTR) or treated by 50 µM FSK for 14 days. (a) Cells were then observed by phase contrast microscopy, allowing to distinguish refractive bright/white BC, indicated by white arrows on phase-contrast microscopic pictures; white bar = 50 µm. BC number (b) or area (c) were next determined by image analysis, as indicated in Methods. Data shown are representative (a) or are the means ± SEM (b, c) of 4 independent assays. (b, c) *, $p<0.05$ when compared to control cells.

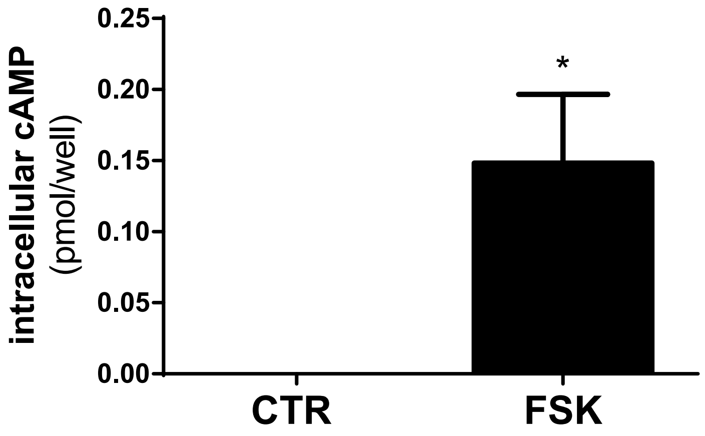

Supplementary Fig. S3. Induction of intracellular cAMP levels in HepaRG cells exposed to FSK.

HepaRG cells seeded in 96 well-plates were either exposed to 0.1 % (vol/vol) DMSO (control/CTR) or treated by 50 µM FSK for 15 min. Intracellular cAMP levels were next determined as indicated in Methods. Data are the means ± SEM of 3 independent assays. *, $p<0.05$ when compared to control cells.

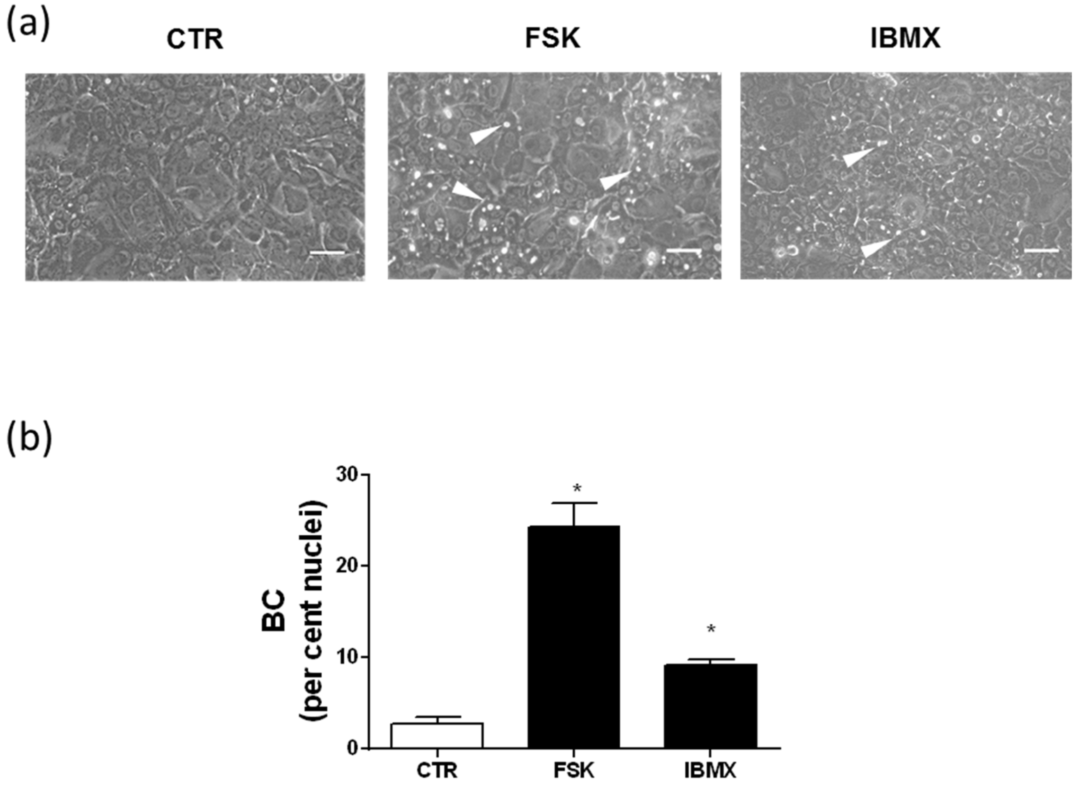

Supplementary Fig. S4. Effect of the cyclic nucleotide phosphodiesterease inhibitor IBMX on BC formation in HepaRG cells.

(a, b) HepaRG were either exposed to 0.1 % (vol/vol) DMSO (control/CTR) or treated for 72 h by 50 µM FSK or 200 µM IBMX. (a) Cells were then observed by phase contrast microscopy, allowing to distinguish refractive bright/white BC, indicated by white arrows on phase-contrast microscopic pictures; white bar = 50 µm. (b) BC numbers were next quantified by image analysis, as indicated in Methods. *, $p<0.05$ when compared to control cells.

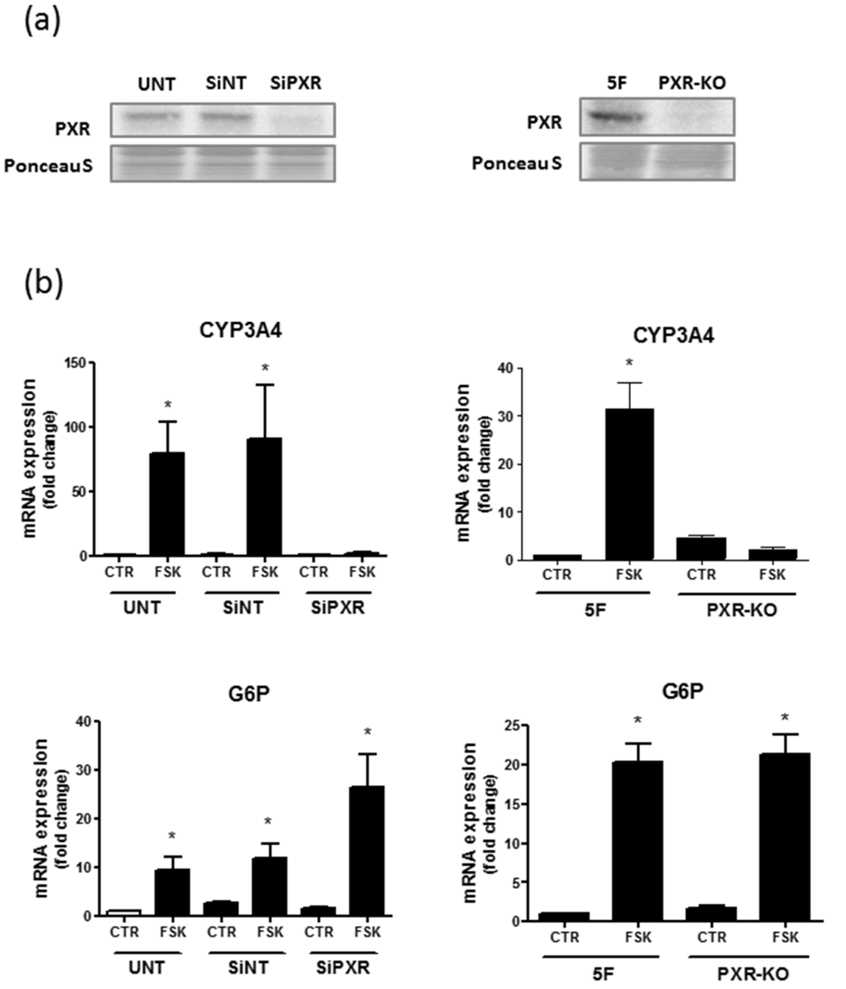

Supplementary Fig. S5. Functional characterization of PXR silencing in HepaRG cells.

(a) Expression of PXR in untransfected (UNT), and SiNT- and SiPXR-transfected HepaRG cells as well as in PXR-KO and control F5 HepaRG clone cells was determined by Western-blotting. Data shown are representative of three independent assays; full length blots are shown in Supplementary Fig. S6. (b) Cells were either exposed to 0.1 % (vol/vol) DMSO (control/CTR) or treated by 50 µM FSK for 72 h. CYP3A4 and G6P mRNA expression were then determined by RT-qPCR. Data are expressed as fold change comparatively to untransfected/untreated control HepaRG cells or to untreated control F5 HepaRG cells; they are the means ± SEM of at least 4 independent assays. *, $p<0.05$ when compared to control untreated counterparts.

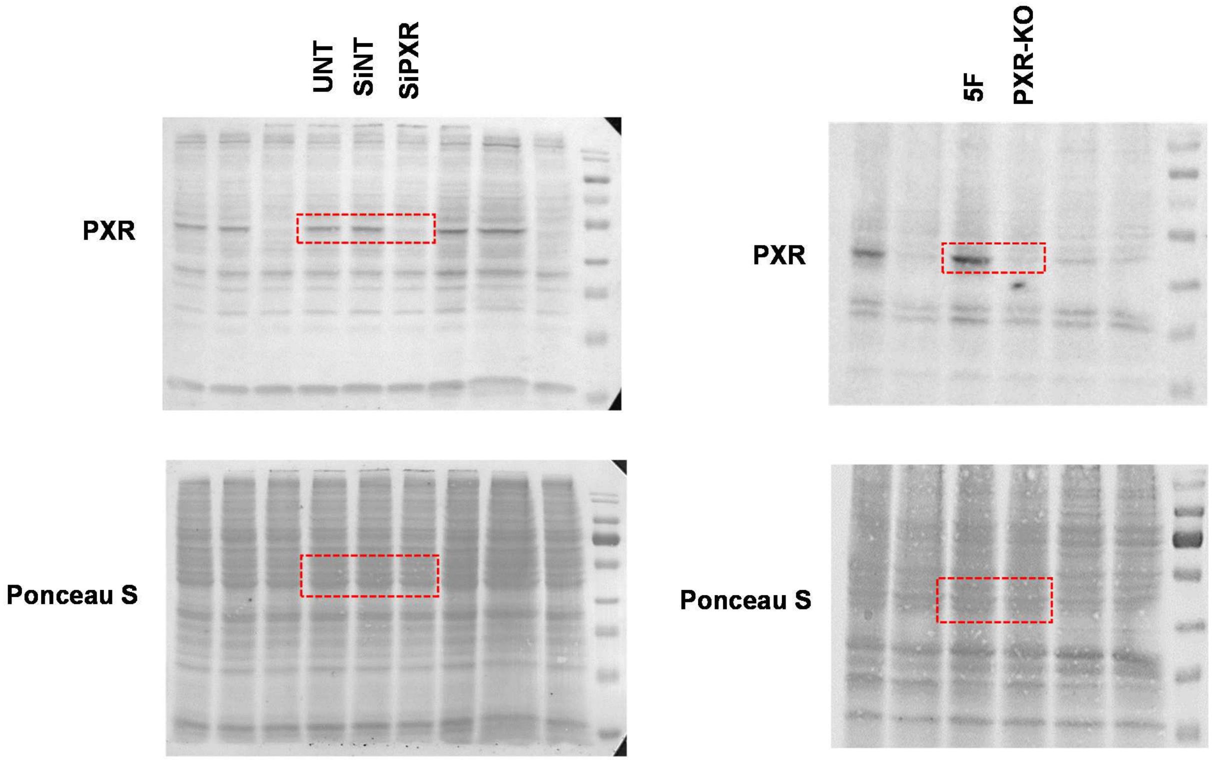

Supplementary Fig. S6. Full lengths blots for Western-blots shown in Supplementary Fig. S5a. Red dotted lines indicate the cropping locations.
